# Effective, but Safe? Physiologically Based Pharmacokinetic (PBPK)-Modeling-Based Dosing Study of Molnupiravir for Risk Assessment in Pediatric Subpopulations

**DOI:** 10.1021/acsptsci.4c00535

**Published:** 2024-11-27

**Authors:** Sarang Mishra, Katharina Rox

**Affiliations:** † Department of Chemical Biology, Helmholtz Centre for Infection Research, 38124 Braunschweig, Germany; ‡ German Centre for Infection Research (DZIF), Partner Site Hannover-Braunschweig, 38124 Braunschweig, Germany

**Keywords:** Molnupiravir, NHC, PBPK, pediatric, dose estimation, toxicity

## Abstract

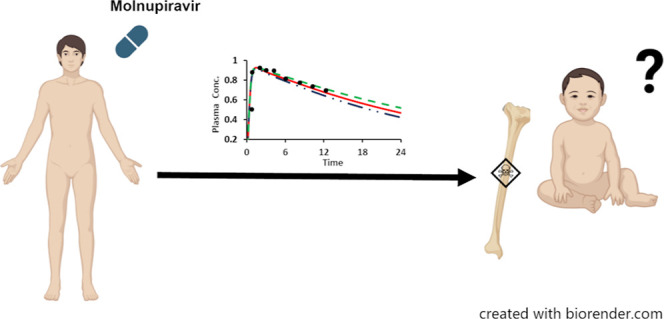

Despite the end of COVID-19 pandemic, only intravenous remdesivir was approved for treatment of vulnerable pediatric populations. Molnupiravir is effective against viruses beyond SARS-CoV-2 and is orally administrable without CYP-interaction liabilities but has a burden of potential bone or cartilage toxicity, observed at doses exceeding 500 mg/kg/day in rats. Especially, activity of molnupiravir against viruses, such as Ebola, with high fatality rates and no treatment option warrants the exploration of potentially effective but safe doses for pediatric populations, i.e., neonates (0–27 days), infants (1–12 months), and children in early childhood (1–12 years). The bone and cartilage toxicity risk for these populations based on the preclinical results has not been systematically investigated yet. Using physiologically based pharmacokinetic (PBPK) modeling, we developed adult PBPK models for doses ranging from 50 to 1200 mg with minimal parameter optimization because of incorporation of CES1, a carboxylesterase. Therein, CES1 served as the main driver for conversion of molnupiravir to its active metabolite *β-d-N4*-hydroxycytidine (NHC). By incorporation of the ontogeny of CES1 for pediatric populations, we successfully developed PBPK models for different doses ranging from 10 to 75 mg/kg. For molnupiravir, efficacy is driven by the area under the curve (AUC). To achieve a similar AUC to that seen in adults, a dose of around 28 mg/kg BID was necessary in all three investigated pediatric subpopulations. This dose exceeded the safe dose observed in dogs and was slightly below the toxicity-associated human equivalent dose in rats. In summary, the pediatric PBPK models suggested that an efficacious dose posed a toxicity risk. These data confirmed the contraindication for children <18 years.

## Introduction

In May 2023, the end of the COVID-19 pandemic was declared, though more than 776 million infections and 7 million deaths had been reported worldwide by October 2024.^1^ Albeit the end of the pandemic, it was the beginning of the endemic phase of SARS-CoV-2.^2−5^ Whereas vaccination has been proven to be an effective measure,^6^ antivirals are still needed, in particular to control and eradicate (re)infections in case of vaccination breakthroughs and vulnerable, high-risk patient populations,^7^ such as elderly, immuno-compromised, and pediatric subpopulations with underlying health conditions. To date, apart from antibody-therapy, several direct-acting antivirals have been approved, such as remdesivir and molnupiravir, both interacting with the RNA-dependent RNA-polymerase as well as M^pro^ inhibitors.^8^ While remdesivir is administered intravenously, making it challenging in the outpatient setting and for pediatric populations, other direct-acting antivirals are approved for peroral therapy. While nirmatrelvir, as a first-generation M^pro^ inhibitor, contained ritonavir as a “booster” to block CYP3A4-mediated clearance,^9^ second-generation M^pro^ inhibitors are metabolically stable.^10,11^ So far, the “ritonavir boost” has resulted in contraindications due to its CYP3A4-mediated drug–drug interaction potential.^7^ By contrast, molnupiravir is perorally bioavailable and does not bear a drug–drug interaction potential.

**Figure 1:**
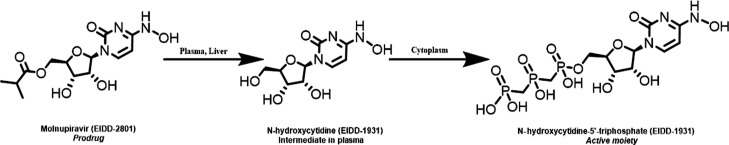
Schematic conversion of molnupiravir toward NHC-TP. Conversion of molnupiravir into its active form (NHC-TP) via NHC. Molnupiravir is rapidly hydrolyzed in plasma to NHC, which is taken up by host cells and further metabolized to the triphosphate form.

Molnupiravir (EIDD-2801) is an orally active prodrug of the ribonucleoside analogue β-d-N4-hydroxycytidine (NHC; EIDD-1931) that was found effective against SARS-CoV-2 in multiple clinical trials,^12^ resulting in emergency use authorization for treatment of COVID-19 by MHRA, US FDA in 2021,^13^ though, to date, not by EMA.^14^ Moreover, it is not only effective against SARS-CoV-2 but also against a wide range of viruses including the Ebola virus,^15^ influenza virus, respiratory syncytial virus,^16^ chikungunya virus,^17^ hepatitis C virus,^18^ and norovirus.^17^ Molnupiravir is rapidly cleaved in plasma toward NHC. NHC, in turn, is rapidly taken up by host cells, where it is converted to the active NHC-triphosphate (NHC-TP) (Figure 1). Then, NHC-TP is eliminated from the body via metabolism to pyrimidines, like cytidine or uridine, mixing with the endogenous nucleoside pool.^12^ NHC-TP itself acts as a competitive substrate for the viral RNA-dependent RNA polymerase to get integrated into the viral RNA, ultimately leading to a viral error catastrophe.^19−21^ The PK parameters of molnupiravir are not fully understood yet because of rapid conversion of the prodrug into NHC and, subsequently, into NHC-TP. Despite a decrease in the maximal concentration (*C*_max_) by the ratio of 1.6 and an increase of the time to maximal concentration (*T*_max_) from 1.0 to 3.0 h, the total exposure, expressed as area under the curve (AUC_0–∞_), is not impacted by concomitant food consumption.^22^ Moreover, preclinical studies in rats at doses exceeding 500 mg/kg/day have raised the concern of bone and cartilage toxicity, leading to a contraindication for children younger than 18 years.^23^

**Figure 2:**
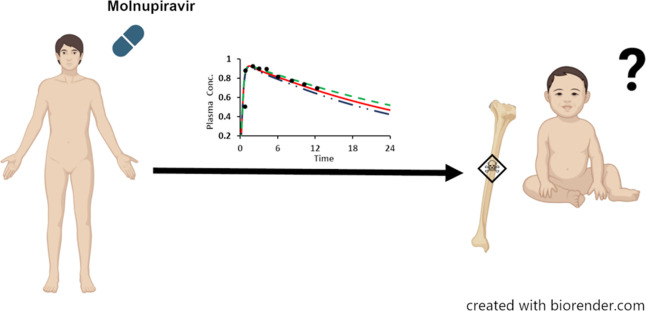
Schematic representation for assessment of a safe but effective dose from adults to children. A safe, but effective dose was modeled using the information from PK data to estimate a safe dose in pediatric populations bearing in mind the risk for bone and cartilage toxicity. The graphic was created with https://www.biorender.com.

Generally, children present milder symptoms from COVID-19 than elderly or adults. However, some vulnerable pediatric subpopulations, such as children with comorbidities, might still develop severe disease requiring treatment.^24^ Typically, this consists inter alia of oxygen therapy, but also, antiviral treatment is used, such as remdesivir intravenously or ritonavir-boosted nirmatrelvir.^25,26^ Bearing in mind that molnupiravir has been proven effective against a diverse set of viruses, such as Ebola virus in a mouse infection study,^15^ we were interested to explore whether there might be an effective dose, which is at the same time safe for children as bone and cartilage toxicity has been observed in preclinical species (Figure 2).^12^

Frequently, pediatric populations have a different expression profile of metabolizing enzymes, resulting in different PK behavior from that in adults. Thus, mechanistic modeling approaches, like physiologically based pharmacokinetic (PBPK) modeling, have been shown to be an effective tool for predicting effective dosage regimens in pediatric populations.^27−29^ PBPK modeling is an alternative to the allometrically based dose adjustment for pediatric populations by employing a mathematical framework, integrating not only the drug’s physiochemical properties but also organism-specific age-scalable physiological properties, like organ volume, density, blood flow, and enzyme expression in different organs. A similar approach has been employed for pediatric dose predictions for remdesivir recently.^28^

The aim of this study was to develop PBPK models of molnupiravir and its active form NHC for adults and deploy the PBPK model to explore consequences on concentration–time profiles for three different pediatric subpopulations, i.e., neonates, infants in early childhood, and children (up to the age of 12 years). The exploration of dosages in pediatric subpopulations took the differences in the metabolizing enzymes as well as physiology compared with adults into account. Then, it was assessed whether there was a safe and efficacious dose conferring a similar effect to that seen in adults.

## Results

Incorporation of esterase as a metabolizing enzyme allows PBPK model development for adults with minimal parameter optimization.

First, for the construction of the adult molnupiravir-NHC PBPK model, the physicochemical properties were incorporated. Moreover, ADME in vitro assays were performed to allow a better estimation of in vivo PK behavior. In particular, the plasma stability and the metabolic stability assays were used to derive information on the half-life of molnupiravir and the subsequent generation of NHC. These assays showed that molnupiravir degraded rapidly in plasma toward NHC, whereas NHC was stable in plasma. In the microsomal metabolic stability assay, NHC had an intrinsic clearance of around 11.6 μL/min/mg protein, whereas the clearance of molnupiravir was much higher at around 40 μL/min/mg protein, suggesting that the major conversion from molnupiravir to NHC takes place in plasma (Table 1). This information was taken into account during parameter optimization. As molnupiravir degraded rapidly in plasma, a slightly higher concentration of molnupiravir was used for the plasma protein binding assay to allow a sufficient remaining amount of molnupiravir for detection after the 2 h incubation time. Both, molnupiravir and NHC had a low plasma protein binding of around 38% and 36%, respectively. The determination of the log *P* value yielded information for calculation of the partition coefficients. Whereas molnupiravir had a slightly higher log *P* value, the log *P* value for NHC suggested no preference above aqueous or lipid phases. Finally, as a peroral PBPK model was developed, the permeability data based on the PAMPA assay results for molnupiravir provided information that moderate permeability can be expected. This value was used as a basis for the subsequent parameter optimization. As NHC is further converted to NHC-TP, which is cleared via the endogenous nucleoside pool, renal clearance as well as hepatic clearance, indicated by our in vitro data, is not involved in clearance. Hence, the GFR ratio was adjusted to one (Table 1).

**Table 1:** Physicochemical Parameters Used for the Molnupiravir-NHC PBPK Model

properties	Molnupiravir	NHC
MW (g/mol)	329.31^34^	259.08^34^
log *P*	0.46^34^	0.10^b^
plasma protein binding (%)	38.08^b^	38.84^b^
microsomal metabolic stability (Cl_int_ [μL/min/mg protein]; in vitro)	40.6^b^	11.6^b^
plasma stability (*t*_1/2_ [min]; in vitro)	<15^b^	>240^b^
permeability (PAMPA, [10^–6^ cm/s])	3.1	
p*K*_a_	2.2, 10.2, 12.0^34^	12.30
solubility [mg/mL]	41^21^	21^21^
hepatic clearance (CES1) [1/min]	15.13^a^	
plasma clearance (CES1) [1/min]	325.26^a^	
unspecified hepatic clearance [1/min]		2.36^a^
specific intestinal permeability [cm/min]	0.000186^b^	
blood/plasma ratio	0.48^c^	0.45^c^
partition coefficient	PK-Sim standard	PK-Sim standard
cellular permeability	PK-Sim standard	PK-Sim standard
GFR ratio	1	1
formulation	Weibull	
dissolution time (*t*_1/2_ [min])	10^a^	
dissolution shape	0.59^a^	

a Optimized parameter.

b Data from in vitro studies.

c PK-Sim predicted.

**Figure 3:**
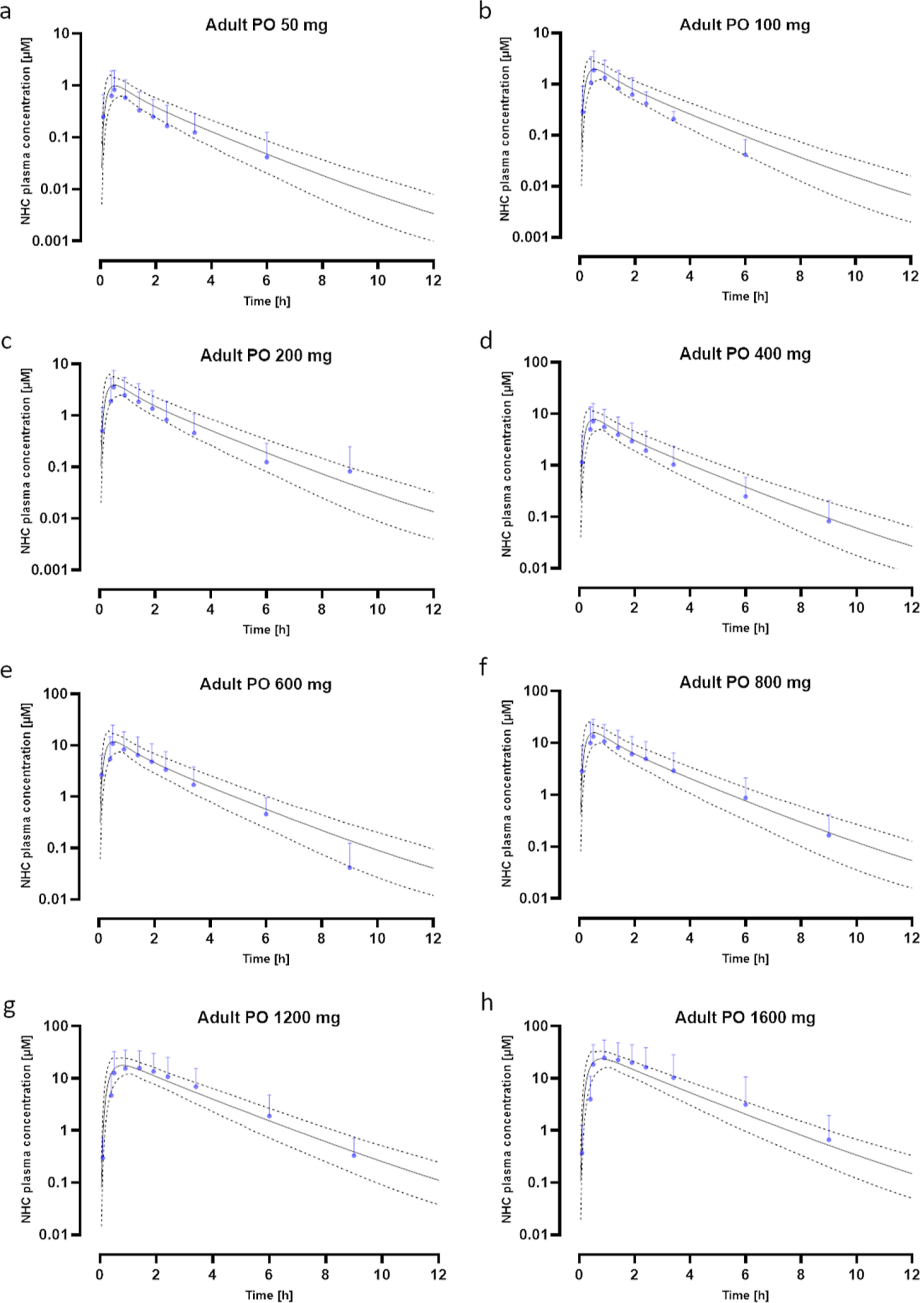
Molnupiravir-NHC PBPK human models for ascending single doses. The continuous line represents the mean simulated plasma concentration of NHC. The dashed lines represent the 5–95% CI. The blue dots with error bars indicate the observed plasma concentrations. All of the observed data are within the CI of the simulated curves. Plasma concentrations of NHC were simulated for oral solutions (a–e) of 50 mg (a), 100 mg (b), 200 mg (c), 400 mg (d), and 600 mg (e) as well as for formulation as a capsule (f–h) for 800 mg (f), 1200 mg (g), and 1600 mg (h).

As molnupiravir is a prodrug, it takes advantage of an easily cleavable ester bond targeted by ubiquitous esterases. Therefore, we selected CES1 as the esterase, present at high abundance in liver and plasma, in our model as the representative metabolizing enzyme for conversion of molnupiravir toward NHC. Next, we employed clinical data from a phase I study with molnupiravir at 50 mg single dose and optimized missing parameters by the Monte Carlo algorithm to fit the simulation to the concentration–time profile observed during clinical testing.^22^ Mainly hepatic clearance and plasma clearance were optimized based on the in vitro determined parameters (Table 1), resulting in only a few optimization rounds. Finally, observed data after 50 mg peroral single dose fit well to the simulation and were within the 95% confidence interval (CI) (Figure 3a). The AUCR as well as the *C*_max_R depicted a slight underprediction with the model of around 20% (Table 2), which is still in an acceptable range.

**Table 2:** Comparison of AUCR and *C*_max_R for Single-Dose PBPK Models

dose (mg)	AUC (ng/mL × *h*)		AUCR	*C*_max_ (ng/mL)		*C*_max_R
	predicted	observed		predicted	observed	
50	361.95	444.22	0.8	214.19	255.01	0.8
100	888.43	769.06	1.2	510.01	485.38	1.1
200	1776.87	1606.63	1.1	1020.02	921.02	1.1
400	3523.88	3450.67	1.0	2040.05	1863.45	1.1
600	5330.60	5376.29	1.0	3060.07	2784.47	1.1
800	7107.46	7694.52	0.9	4080.08	3437.75	1.2
1200	10643.55	14091.83	0.8	4545.37	4069.61	1.1
1600	14191.11	21964.72	0.6	6060.36	6393.57	0.9

Next, we aimed to assess the predictability at the ascending doses. With different ascending doses as observed data at hand,^22^ the single-dose molnupiravir-NHC PBPK model was extrapolated to 100 mg, 200 mg, 400 mg, and 600 mg peroral single doses (Figure 3b–e). For the 100 mg peroral single dose, observed data were in the lower range of the 95% CI (Figure 3b). The AUCR showed a slight overprediction for the AUC of around 20%, whereas the *C*_max_R only had a slight overprediction of 10% (Table 2). Whereas some observed data points for the 200 mg peroral single dose were in the lower range of 95% CI (Figure 3c), observed data points for the 400 and 600 mg single doses fit well with the simulation (Figure 3d,e). This was also reflected in the AUCR and *C*_max_R values (Table 2). Additionally, the simulation for the 800 mg single dose fit well with the observed data (Figure 3f), which was in line with the AUCR and *C*_max_R values (Table 2). Based on the comparison of AUC of the respective simulations as well as of the *C*_max_, dose linearity was observed for doses up to 800 mg, which also complied with the observed data (Figure S1).

Starting from the 1200 mg single dose, molnupiravir was given as a capsule formulation. To account for the different dissolution profiles, a Weibull first-order absorption kinetic was used. Moreover, the 1200 mg observed data were used to optimize parameters for the dissolution profile of the capsule using the Monte Carlo algorithm. No further optimizations in terms of clearance were performed; instead, the PBPK model based on the optimization with the 50 mg dose was employed. The input parameters accounting for the different formulation are found in Table 1. After optimization for the capsule formulation, the simulated concentration–time profile for NHC for the 1200 mg single dose fit well with the observed data, which were within 95% CI (Figure 3g). Moreover, the AUCR showed a slight underprediction of 20% of the overall exposure with a slight overprediction of 10% of the *C*_max_ compared to observed data (Table 2). Despite the observed data being in the upper range of the 95% CI for the 1600 mg simulation (Figure 3h), analysis of the overall exposure showed that it was largely underpredicted, whereas the *C*_max_R was in an acceptable range (Table 2). Furthermore, for both 1200 mg and 1600 mg dose, a delayed *C*_max_ was observed compared to that for the doses administered as solution. This suggests that the delayed *C*_max_ was attributed to the different formulations, as hypothesized by Painter and colleagues.^22^ The analysis for dose linearity for *C*_max_ showed that *C*_max_ continued to be linear with increasing dose, but this was not observed for AUC over the entire dose range (Figure S1, Table 2). In line with this, the AUC increase was linear for observed data from 1200 to 1600 mg (Figure S1), whereas it was not linear to doses lower than 800 mg.

Next, we were interested in evaluating the performance of our PBPK models using the capsule formulation at lower doses (from 50 to 800 mg) for multiple dose administrations at a dosing interval of q 12 h for 5.5 days. The observed data, which were available for the first and last administered dose from the study conducted by Painter and colleagues,^22^ fit well for the entire dose range from 50 to 800 mg within the 95% CI of the simulated curves (Figure S2). However, as already seen for the single dose capsule formulation at 1200 mg and 1600 mg, a decreased *C*_max_ and a delayed *T*_max_ were observed. A closer examination of the fit was performed by determination of the AUCR and *C*_max_R values (Table 3). It was detected that the ratios for *C*_max_R values were only deviating minimally for doses up to 600 mg BID. A similar observation was made for AUCR (Table 3). At the dose of 800 mg BID, the PBPK model was slightly underpredicting the AUC and the *C*_max_ compared to the observed data. Compared to the 800 mg dose administered as solution, it was observed that the overall exposure also decreased when the capsule formulation was used (Tables 2 and 3). The two PBPK models for the different formulations captured that.

**Table 3:** Comparison of AUCR and *C*_max_R for Multiple Dose PBPK Models

dose (mg)	AUC (ng/mL × *h*)		AUCR	*C*_max_ (ng/mL)		*C*_max_R
	predicted (total)	observed 1st dose | lastdose		predicted (total)	observed 1st dose | last dose	
50	4912.08	427.62 | 415.08	1.06	189.94	231.56 | 182.3	0.92
100	9824.17	853.89 | 938.6	1.00	379.89	369.62 | 413.02	0.97
200	19648.23	1566.09 | 1668.9	1.10	759.77	655.79 | 710.56	1.11
300	29472.64	2995.61 | 2960.25	0.90	1139.65	1230.28 | 1016.0	1.01
400	39296.59	3720.4 | 3632.55	0.97	1519.52	1461.72 | 1452.91	1.04
600	58944.48	6374.4 | 7403.18	0.78	2279.28	1839.33 | 2108.48	1.15
800	50566.73	8288.8 | 8155.42	0.56	1955.37	2659.51 | 2881.23	0.71

Different CES1 metabolizing capacities result in distinct concentration–time profiles for pediatric subpopulations compared to adults.

As the adult PBPK models exhibited a good strength of prediction over a dose ranging from 50 to 1200 mg by incorporation of CES1 as a metabolizing enzyme in plasma and liver, we set out to scale the adult PBPK models toward three different pediatric subpopulations. The prediction of achievable plasma concentrations of NHC was envisaged as molnupiravir has not been assessed in children due to safety concerns.^23^ First, the pediatric PBPK models for three age groups, i.e., neonates, infants, and children in early childhood (Tab. S2), were developed by scaling from the adult PBPK model. In brief, the PK-Sim built-in age-related physiological parameters including plasma protein abundances were employed for the model development for each subpopulation. Moreover, the CES1 ontogeny and expression profile was added for each pediatric subpopulation based on published information.^31^ Since renal clearance is not involved in elimination of molnupiravir and NHC, the GFR ratio was not modified. Furthermore, all remaining parameters were kept equal to those of the adult PBPK models. For the pediatric population, a ratio of 1:1 for male and females was used for all corresponding age ranges. Additionally, as children might not have the capabilities to take capsules, oral solution as the formulation was used. Moreover, the adult PBPK models with the solution formulation predicted plasma levels well, giving confidence for the development of the pediatric models.

**Figure 4:**
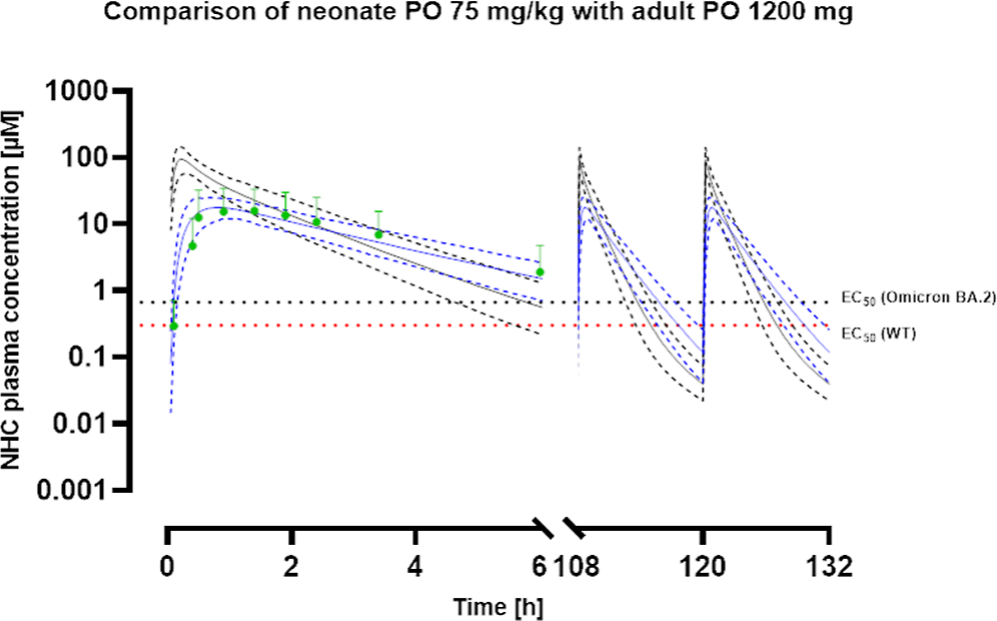
Concentration–time profiles for neonates at 75 mg/kg BID compared to adults at 1200 mg BID. NHC plasma concentration–time profiles for 75 mg/kg of BID (black) in neonates and 1200 mg of BID (blue) in adults over 5.5 days. Mean observed data as well as error bars for adults are shown in green. The continuous lines represent the predicted mean plasma concentrations, whereas the lower and upper dashed lines depict the 95% CI. The EC_50_ for the omicron (black) variant and the wildtype (red) are shown as dashed lines.

**Figure 5:**
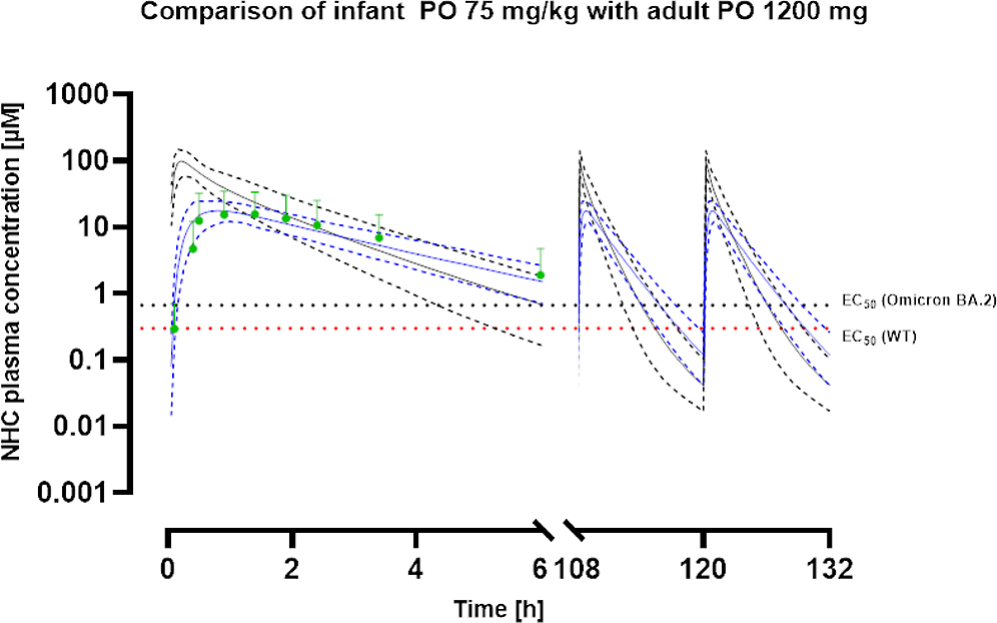
Concentration–time profiles for infants at 75 mg/kg BID compared to adults at 1200 mg BID. NHC plasma concentration–time profiles for 75 mg/kg of BID (black) in infants and 1200 mg of BID (blue) in adults over 5.5 days. Mean observed data as well as error bars for adults are shown in green. The continuous lines represent the predicted mean plasma concentrations, whereas the lower and upper dashed line depict 95% CI. The EC_50_ for the omicron (black) variant and the wildtype (red) are shown as dashed lines.

**Figure 6:**
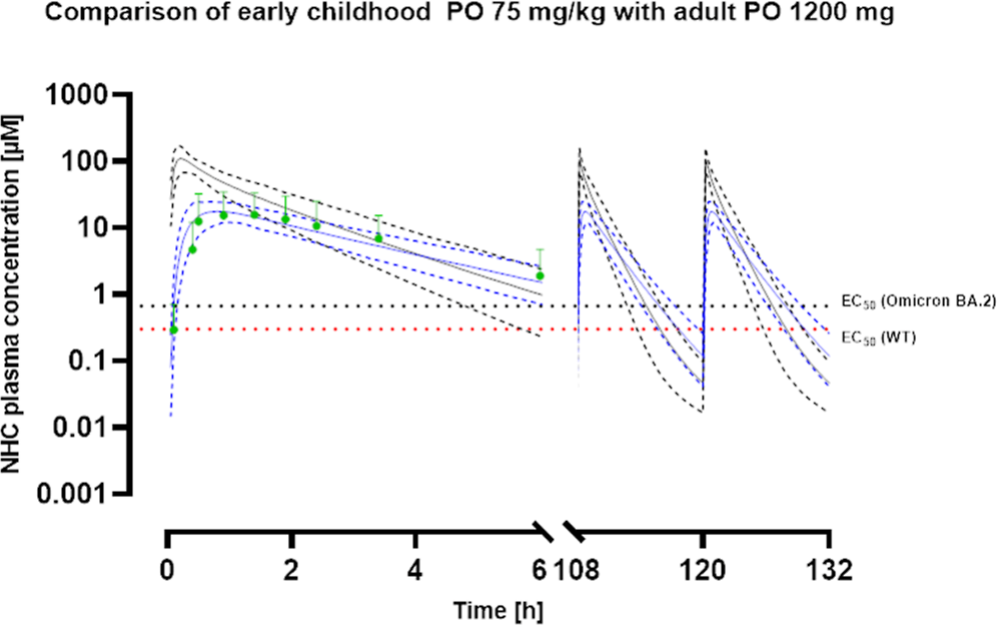
Concentration–time profiles for children in early childhood at 75 mg/kg BID compared to adults at 1200 mg BID. NHC plasma concentration–time profiles for 75 mg/kg of BID (black) in children in early childhood and 1200 mg of BID (blue) in adults over 5.5 days. Mean observed data as well as error bars for adults are shown in green. The continuous lines represent the predicted mean plasma concentrations, whereas the lower and upper dashed line depict 95% CI. The EC_50_ for the omicron (black) variant and the wildtype (red) are shown as dashed lines.

Similar to the adult PBPK models, dosing over 5.5 days was simulated at an interval of q 12 h. The doses of 10 to 75 mg/kg q 12 h were used to explore achievable exposure as well as *C*_max_ levels. Even with the same doses expressed as mg/kg per age group, children in early childhood achieved the highest *C*_max_ levels and highest exposures, whereas neonates had lower exposures and slightly lower *C*_max_ levels (Table 4). The concentration–time profiles for individual representatives of the respective subpopulation showed that dose linearity was observed for all three subpopulations for doses ranging from 10 to 28 mg/kg q 12 h (Figures S3–S5). Interestingly, the *T*_max_ increased at higher doses and with age, as seen for children in early childhood compared to neonates or infants (Figures S3–S5). This effect partially vanished when populations were used for each pediatric subgroup (Figures 4, 5, and 6). As for neonates, a population ranging from 0 to 27 days of age was used, and higher variability was seen, resulting in a lower mean *T*_max_ (Figure 4). The same was observed for the population of infants ranging from 1 month to 12 months of age (Figure 5) as well as for children in early childhood ranging from 1 to 12 years (Figure 6). Compared to the 800 mg BID dose for adults, corresponding to roughly 11 mg/kg, all three pediatric subpopulations showed similar *C*_max_ values (Table 4). However, exposures were much lower than those for adults. Within the three pediatric subpopulations, neonates showed the lowest level of exposure. To achieve a similar exposure to that in adults at the prescribed dose of 800 mg BID, a dose of around 28 mg/kg BID was necessary for all three subpopulations (Table 4).

**Table 4:** Predicted PK Parameters for Adult and Pediatric Models at Different Oral BID Doses for 5.5 Days

dose [mg/kg]	neonates		infants		early childhood		adult (800 mg)	
	AUC [μM × *h*]	*C*_max_ [μM]	AUC [μM × *h*]	*C*_max_ [μM]	AUC [μM × *h*]	*C*_max_ [μM]	AUC [μM × *h*]	*C*_max_ [μM]
10	136.79	12.95	143.6	13.65	176.66	15.19	352.29	12.32
14	191.51	18.13	201.05	19.11	247.32	21.27		
28	383.02	36.25	402.09	38.22	494.65	42.53		
50	683.97	64.72	718.02	68.25	883.2	75.95		
75	1025.95	97.1	1077.04	102.38	1324.95	113.93		

### Effective Doses for Pediatrics Bear a Toxicity Risk

Analogously to the PK/PD index in antibacterial drug development,^35^ several concepts are conceivable for antivirals predicting which PK behavior is necessary to get a PD effect. For ritonavir-boosted nirmatrelvir, it was aimed to achieve high concentrations for a maximal time over the in vitro determined EC_50_.^9^ Therefore, we calculated the time window for concentrations of NHC over EC_50_ for the prescribed dose of 800 mg BID. We deployed the EC_50_ value of 0.67 μM (omicron variant) of NHC (Table S3).^36^ The adult PBPK model suggested a time of 8.7 h over EC_50_ for the recommended dose of 800 mg BID. The time over EC_50_ for neonates, infants, and children in early childhood at 10 mg/kg BID was 3.15, 3.25, and 3.7 h, respectively (Table S4). When extrapolating the dose for the pediatric subpopulations further toward 75 mg/kg BID, the time increased to 7.0, 7.3, and 7.9 h, respectively (Table S4). Still, it was much lower than that observed for adults at the prescribed dose.

Another concept apart from time over EC_50_ is the total exposure in relation to EC_50_. This has been recently proposed for driving the PD effect of molnupiravir and NHC.^12,37^ As the 800 mg BID dose was achieving a near-maximal effect during clinical testing,^37^ the exposure of around 350 μM × *h* was used as target AUC needed for pediatric populations. For all three subpopulations, a dose of around 28 mg/kg BID was required to achieve a similar exposure to that observed for adults (Table 4).

So far, molnupiravir is contraindicated in children younger than 18 years due to the risk of bone and cartilage toxicity. That toxicity was observed in rats dosed with more than 500 mg/kg/day over three months in rats, whereas no toxicity was observed in dogs at 50 mg/kg/day over a time frame of 14 days.^23^ Nair and Jacob provided scaling factors for allometric scaling from rat to human.^38^ According to these scaling factors, the 500 mg/kg/day dose in rats corresponds to around 80 mg/kg/day in humans. Similarly, the nonobserved toxicity dose in dogs of 50 mg/kg/day would correspond to a human equivalent dose of 27 mg/kg/day. To achieve a similar time over EC_50_ as for adults, pediatric doses of around 150 mg/kg/day are required, exceeding the no observed effect level by several magnitudes (Table S4). Additionally, doses of 75 mg/kg exhibited an increased *C*_max_ and exposure compared to an increased dose of 1200 mg of BID in adults (Figures 4, 5, and 6). The exposure has been identified as the relevant PD driver for molnupiravir.^37^ To achieve the same exposure as in adults, a dose of 28 mg/kg, i.e., 56 mg/kg/day is required to achieve an effect based on the current assumptions for the PD driver. This dose is above the no observed effect level in dogs and slightly below the toxicity level in rats.

## Discussion

Molnupiravir is contraindicated in children younger than 18 years due to the potential of bone and cartilage toxicity.^23^ However, to date, no systematic evaluation has been performed. As it is not ethically feasible to administer a drug associated with a toxicity risk for growing bones to children, we set out to explore the potential risk based on a modeling and simulation exercise. Our motivation was driven by the fact that molnupiravir does not harbor a drug–drug interaction potential, is orally administrable,^39^ and has activity against different viruses with high fatality rates,^15,16^ for which treatment options are not yet available. Additionally, the Pediatric Infectious Diseases Society of America recently emphasized that a data basis for molnupiravir treatment for COVID-19, particularly for children younger than 12 years, is still lacking.^25^

For molnupiravir, it is known that it is cleaved rapidly to NHC in plasma. No reports mention which enzyme is responsible. By contrast, for remdesivir, harboring a similar ester functionality to molnupiravir, it has been assessed in vitro that CES1, carboxylesterase, is responsible for cleavage.^26^ Thus, we assumed that CES1 might be equally responsible for conversion from molnupiravir toward NHC. By incorporation of CES1 as the main metabolizing enzyme, we were able to predict the clinically observed concentration–time profiles over a range of 50 to 1600 mg within a 95% CI. Doses at 1200 and 1600 mg in adults using the capsule formulation exhibited a lower *C*_max_, which was not well captured by the simulation. A similar observation was made for the 800 mg multiple dose simulation using the capsule formulation. The observed data gave higher AUCs than the corresponding simulations. This effect was not observed for the capsule formulation at doses lower than 800 mg. This suggests that the capsule formulation at higher doses contributed to an increased absorption, which was not reflected by our simulation.

Solutions are preferred over capsules for pediatric populations. Our model for 800 mg with the solution as a formulation had a good strength of predictability. Thus, we used this modeling framework to predict pediatric doses. For remdesivir, PBPK modeling as well as allometric scaling for pediatric populations has been performed.^28,40^ In PBPK models, the pediatric dose estimation for remdesivir was either weight-based or exposure-based to achieve exposures similar to the recommended adult dosing regimen.^28,40^ A similar strategy was employed in this study. It is known for molnupiravir that the effect is driven by exposure.^37^ Therefore, we aimed to achieve a similar exposure in different pediatric subpopulations equivalent to the recommended dose of 800 mg of BID in adults. Due to unavailability of clinical observed data for pediatrics, the plasma concentration–time profile predictions were conducted for doses ranging from 10 to 75 mg/kg BID to attain similar profiles as seen in adults. The incorporation of CES1 for the three different pediatric subpopulations showed that exposures were much lower, albeit similar *C*_max_ values. This is attributable to the gradually increasing CES1 activity during the pediatric age. The pediatric models were not able to achieve effective NHC plasma concentrations due to overall lower levels of CES1 in neonates, followed by infants and children in early childhood of around 19, 43, and 76% of adult CES1 level,^31,41−43^ respectively. Presumably, this limited the conversion of molnupiravir to NHC. In line with the simulated data for the three pediatric populations, a dose of around 28 mg/kg of BID was needed to achieve similar exposures to that in adults. Moreover, doses of 75 mg/kg BID in neonates, infants, and children in early childhood do not suffice to result in the same time over EC_50_ compared to adults.

It has been shown in preclinical species that high doses of molnupiravir over three months can result in bone and cartilage toxicity. Although toxicity studies in dogs over 14 days did not result in any toxicity flags, molnupiravir is currently contraindicated for pediatric populations.^23^ Bone toxicity parameters are critical for pediatric populations with growing bones and cartilages, as observed in the case of tetracycline deposition in bones and other organs.^44^ Therefore, we deployed the model developed for pediatrics to estimate a potential safe and efficacious dosing level, avoiding bone toxicity. Data from regulatory agencies served as the basis for these calculations. Our data reveal that doses achieving the same exposure as in adults slightly exceed the safe dose in dogs (allometrically scaled to a human equivalent dose). Although the dose needed in pediatrics is below the dose causing toxicity in rats, it is unclear which PK drives toxicity in bones and cartilages. Our data suggest that higher doses in the three different pediatric populations result in higher *C*_max_ values. In case toxicity is not driven by exposure but rather by peak levels, this could aggravate the outcome for pediatrics.

Although we used clinically observed data, the data were digitalized from existing studies. Potentially, this can lead to smaller deviations from the observed data. This could have resulted in slight differences with regard to the evaluation of the goodness of fit. Moreover, a key study limitation is that pediatric observed data are not available and that predictions were only made based on the adult PBPK model validated with the clinical observed PK data. Regarding the CES1 ontogeny profile, data were derived from published studies.^31^ However, only broader ranges of CES1 for different age ranges were retrieved. This might not capture the entire range of CES1 amounts and activities for the different ages used in this study. It is known from CYP enzymes that they mature differently so that the sparse information about CES1 is a limitation. Nevertheless, compared to the pediatric PBPK-modeling study for remdesivir,^28^ more CES1 information was incorporated. This provides more confidence for the predictions in pediatrics. It is not known what PK parameters drove the toxicity in the preclinical species. Therefore, predictions of toxicity for pediatrics can only be made based on total dose. Additionally, it is not known if conversion from NHC toward NHC-TP has a similar velocity to that observed in adults, as this is also an enzyme-dependent process, which might be also altered in children. It has been seen that NHC-TP levels are much higher in cells, even when NHC levels in plasma drop already.^12^ Due to sparse information about converting enzymes and their maturation characteristics in children, NHC-TP levels have not been predicted. In case NHC-TP is the actual driver of toxicity and in case NHC-TP results in higher levels in pediatrics than in adults, toxicity might even occur at lower concentrations in pediatrics. This scenario associates even higher risks with molnupiravir administration in pediatric populations. Furthermore, as NHC-TP is prone to create new variants of concern by mutagenesis through its mechanism of action,^45^ higher NHC-TP levels in children could contribute to an increase in the occurrence of variants of concern.

In summary, the incorporation of CES1 as a metabolizing enzyme allowed PBPK models to be constructed for a broad dose range in adults with minimal need for parameter optimization. Equally, CES1 incorporation for the pediatric subpopulations investigated in this study revealed the consequences on *C*_max_ and AUC values. This called for 2–3-fold higher doses needed to achieve similar efficacy to that in adults. The preclinical data suggested levels at which bone and cartilage toxicity was observed. Our data suggest that doses needed for efficacy in pediatric populations bear a significant toxicity risk. Thus, this risk was not ruled out. Consequently, our study suggests that orally administered molnupiravir is not an option for antiviral treatment in pediatric populations.

## Methods

### ADME Assays

#### Plasma Protein Binding, Plasma Stability, and Metabolic Stability Assay

The plasma protein binding assay, the plasma stability assay, and the metabolic stability assay were performed as described previously.^30^ For all of the assays, NHC and molnupiravir were used. Molnupiravir and NHC were purchased from Merck/Sigma.

#### Experimental Determination of log *P*

The log *P* experiment was performed for molnupiravir and its metabolite NHC. Octan-1-ol and water were saturated 24 h prior to the start of the experiment. Each compound was added to the octan-1-ol phase at a final concentration of 100 ng/mL. Two setups were used: octan-1-ol/water (1:1) and octan-1-ol/water (1:10). After addition of the compound, each phase was shaken at 2000 rpm for 1 h, and then, phases were separated by centrifugation at 13,000 rpm for 5 min at 4 °C. From each sample, 10 μL of water layer and octanol layer was diluted to 100 μL with DMSO for high-performance liquid chromatography tandem mass spectrometry (HPLC-MS/MS) analysis. The log *P* was calculated by Formula 1.
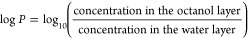
1

#### PAMPA Study

For the PAMPA assay, a PAMP-096 kit from BioAssay Systems was used. Theophyllin, chloramphenicol, and diclofenac were used as low, high, and medium permeability controls, respectively. The assay was conducted as described in the manufacturer’s protocol. For all compounds, 10 mg/mL DMSO stocks were used. Compound concentrations were determined using HPLC-MS/MS. For the HPLC-MS measurements, samples were analyzed using an Agilent 1290 Infinity II HPLC system coupled to an AB Sciex QTrap 6500plus as described below. Permeability was determined as follows in eqs 2 and 3.

Effective permeability (*P*_eff_) was calculated using the following formula
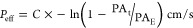
2
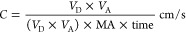
3where the PA_t_ is average peak area of the test compound or permeability standard and PA_E_ is the peak area of the equilibrium standard. The value of C was calculated by the following formula, where donor volume (*V*_D_) is 0.2 cm^3^, acceptor volume (*V*_A_) is 0.3 cm^3^, membrane area is 0.24 cm^2^, and incubation time is 72,360 s.

#### HPLC-MS/MS Analysis

Samples were analyzed using an Agilent 1290 Infinity II HPLC system coupled to an AB Sciex QTrap 6500plus mass spectrometer. LC conditions were as follows: column: Agilent Zorbax Eclipse Plus C18, 50 × 2.1 mm, 1.8 μm; temperature: 30 °C; injection volume: 1 μL per sample; flow rate: 700 μL/min. Samples were collected under the following conditions. Solvents: A: 100% water +0.1% HCOOH; solvent B: 100% ACN +0.1% HCOOH. The gradient was as follows: 99% A at 0 min, 99% A until 1 min, 99–0% A from 1 to 3 min, 99% A until 3.2 min. Mass transitions are depicted in Table S1. Peaks of samples were quantified by using relative quantification. Data analysis was performed by using Multiquant 3.0 software (AB Sciex).

### PBPK Model Development

#### Adult PBPK Model

The adult whole-body PBPK model was developed based on the physiology of an adult European male (age: 30 years, weight: 73 kg) using a middle-out approach. PK-Sim Standard predicted partition coefficient and cellular permeability were utilized in the model (Table 1). Molnupiravir and NHC were used for compound building blocks. Physicochemical parameters were added as outlined in Table 1. As molnupiravir harbors an ester, it was assumed that the ester bond is quickly cleaved in plasma by esterase. Therefore, CES1 as an enzyme was added for metabolism in plasma. Parameters like reference enzyme abundance^31^ and *t*_1/2_ value^32^ of CES1 were updated in the model along with the PK-SIM in-built relative distribution of CES1. Further in vitro parameters were either experimentally determined or derived from the literature as shown in Table 1. PK data were derived from clinical studies^22^ as well as from the FDA fact sheet and the EMA assessment report.^33,34^ Parameters of the PBPK model built with in vitro data were optimized using the clinical human PK data at a 50 mg single dose. The model was then validated at higher doses and two different formulations, capsule and solution, as well as multiple dosing regimens. The capsule formulation was used for the doses of 800, 1200, and 1600 mg. For the capsule, the Weibull dissolution profile was used, and parameters for Weibull were optimized based on the 800 mg observed data. For validation and goodness of fit, the 95% CI was used as well as a description of the AUCR and *C*_max_R. Furthermore, the AUC and the *C*_max_ ratios (AUCR and *C*_max_R) for the predicted and the observed values were determined to understand if predicted data are in line with the observed data.

Next, multiple dose PBPK models for the doses of 50, 100, 200, 300, 400, 600, and 800 mg q 12 h using the capsule formulation were built. Thereby, optimized parameters for the capsule determined with the 1200 mg capsule single dose PBPK model were used (Table 1).

#### Pediatric PBPK Model for Neonates, Infants, and Children in Early Childhood

The adult PBPK model of molnupiravir and NHC was extrapolated by employing PK-Sim built-in scaling of age-dependent physiological and anatomical parameters such as weight, organ volume, body mass index, and plasma protein binding. For the pediatric PBPK models, three different subpopulations were investigated: (a) neonates (0–27 days), (b) infants (28 days −1 year), and (c) children in early childhood (1–12 years) (Table S2). It accounted for the different amounts of CES1 in these three age groups based on literature information.^31^ The GFR ratio was set to one. For the pediatric subpopulations, virtual populations for the entire age range of the respective subpopulation with a 1:1 ratio of females to males were generated using a Monte Carlo simulation. For every subpopulation, 100 individuals were generated. Doses were administered as oral solution from 10 mg/kg q 12 h until 75 mg/kg q 12 h.

#### Software

The PBPK model was developed using PK-Sim software of Open Systems Pharmacology suite, version 11.2 (http://www.open-systems-pharmacology.org). Models were optimized by using sensitivity analysis and parameter optimization based on Monte Carlo algorithms with randomized multiple optimization. GetData Graph Digitizer, version 2.26 (http://getdata-graph-digitizer.com) was used to digitize clinical data from the reported literature. Graphs were generated using Prism 10, Graphpad Inc. (https://www.graphpad.com).

## Supporting Information Available

Mass spectrometric conditions for molnupiravir and NHC; demographic and ontogenic parameters for subpopulations of the study; EC_50_ values of NHC against different SARS-CoV-2 VOC; time over EC_50_ for adult and pediatric models at selected doses; dose linearity comparison for dose correlated with AUC and *C*_max_; simulated and observed concentration–time profiles for NHC after administration of multiple doses; NHC plasma concentration–time profile for neonates at different doses; NHC plasma concentration–time profile for infants at different doses; and NHC plasma concentration–time profile at different doses for children in early childhood (PDF)
