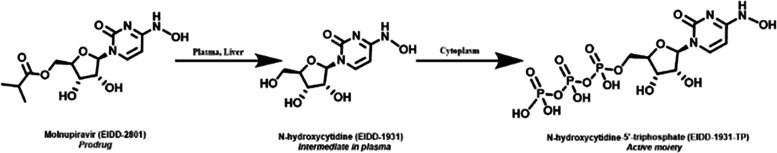# Correction to “Effective, but Safe? Physiologically Based Pharmacokinetic (PBPK)-Modeling-Based Dosing Study of Molnupiravir for Risk Assessment in Pediatric Subpopulations”

**DOI:** 10.1021/acsptsci.5c00053

**Published:** 2025-01-28

**Authors:** Sarang Mishra, Katharina Rox

After publication of the manuscript, we found an error in the structure of the active moiety of molnupiravir as the O atoms were missing in the triphosphate in Figure 1. The corrected structure within Figure 1 is provided.

**Figure 1:**