# Supplementary material for: Effective, but Safe? Physiologically Based Pharmacokinetic (PBPK)-Modeling-Based Dosing Study of Molnupiravir for Risk Assessment in Pediatric Subpopulations
Source: ACS Pharmacol Transl Sci. 2024 Nov 27;7(12):4112–22. doi: 10.1021/acsptsci.4c00535 (PMC11651168; doi:10.1021/acsptsci.4c00535)
Supplement: Supplementary file 1 — pt4c00535_si_001.pdf [file pt4c00535_si_001.pdf]

Effective, but Safe? Physiologically Based Pharmacokinetic (PBPK)-Modeling-Based Dosing Study of Molnupiravir for Risk Assessment in Pediatric Subpopulations

*Sarang Mishra[1], Katharina Rox[1,2]**

[1]*Department of Chemical Biology, Helmholtz Centre for Infection Research, Braunschweig*
[2]*German Centre for Infection Research (DZIF), partner site Hannover/Braunschweig, Braunschweig, Germany*

*Correspondence:

Dr. Katharina Rox, E-mail: katharina.rox@helmholtz-hzi.de, phone: +49 531 6181 4409

Table of content

Supplemental tables 3
Table S1. Mass spectrometric conditions for molnupiravir and NHC 3
Table S2. Demographic and ontogenic parameters for subpopulations of the study 3
Table S3. EC_{50} values of NHC against different SARS-CoV-2 VOC 3
Table S4. Time over EC_{50} for adult and pediatric models at selected doses 3

Supplemental Figures 4
Figure S1. Dose linearity comparison for dose correlated with AUC and C_{max} 4
Figure S2. Simulated and observed concentration-time profiles for NHC after administration of multiple doses. 5
Figure S3. NHC plasma concentration-time profile for neonates at different doses 6
Figure S4. NHC plasma concentration-time profile for infants at different doses 6
Figure S5. NHC plasma concentration-time profile at different doses for children in early childhood 7

Supplemental tables

Table S1. Mass spectrometric conditions for molnupiravir and NHC

COMPOUND	Q1 (DA)	Q3 (DA)	DP (V)	CE (V)	CXP (V)
**CAFFEINE**	195.024	138.0	130.0	25.0	14.0
		110.0	130.0	31.0	18.0
**MOLNUPIRAVIR**	327.921	168.0	-105.0	-22.0	-17.0
		125.9	-105.0	-22.0	-13.0
**NHC**	257.918	167.9	-20.0	-20.0	-15.0
		126.0	-20.0	-20.0	-7.0

Table S2. Demographic and ontogenic parameters for subpopulations of the study

	Neonates	Infants	Early Childhood	Adults
**Age range (years)**	0 - 0.083	0.1 - 1	1 - 6	18 - 60
**Weight range (kg)**	2.7 - 5.7	3.5 - 14	06 -28	65 - 96
**CES1 protein abundance (pmol/mg total protein)**	315.2	722.2 6	1262.1	1664.4

CES1 protein abundance data were derived from Boberg *et al.*, 2016[31]

Table S3. EC_{50} values of NHC against different SARS-CoV-2 VOC

	Antiviral activity, EC_{50} (μM)					
	wildtype	beta	alpha	delta	omicron BA.1 lineage	omicron BA.2 lineage
NHC	0.30	0.48	0.53	0.89	0.28	0.67

EC_{50} data were derived from Wen *et al.*, 2023[36]

Table S4. Time over EC_{50} for adult and pediatric models at selected doses

Dose [mg/kg]	neonates	infants	Early childhood	Adult [800 mg]
**10**	3.15 hours	3.25 hours	3.7 hours	8.7 hours
**75**	7.0 hours	7.3 hours	7.9 hours	

Supplemental Figures

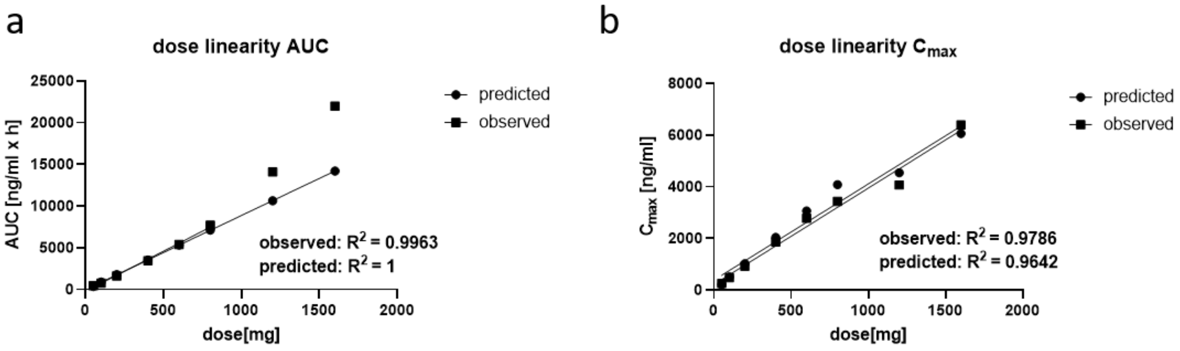

Figure S1. Dose linearity comparison for dose correlated with AUC and C_{max}

Dose linearity was assessed for dose with AUC for predicted and observed data (a) as well as for dose with C_{max} for predicted and observed data (b). Dose linearity for AUC was observed until a dose of 800 mg for observed data with a R^2 of 0.9963 excluding AUC values of 1200 and 1600 mg (a). By contrast, C_{max} was dose-linear for observed and predicted values up to 1600 mg (b).

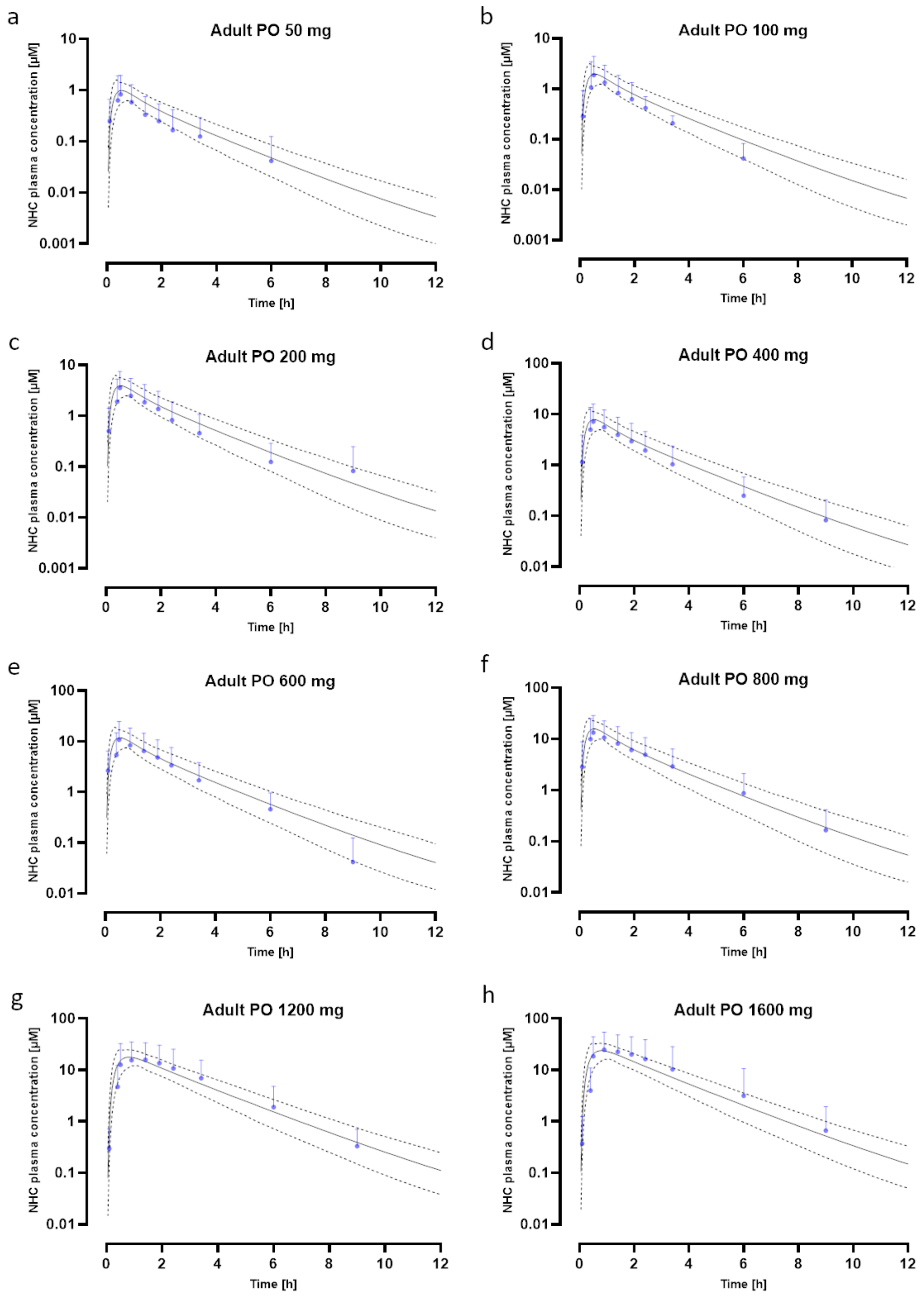

Figure S2. Simulated and observed concentration-time profiles for NHC after administration of multiple doses.

Molnupiravir was administered as capsule over 5.5 days as a q12h dosing scheme. The continuous line represents the mean simulated plasma concentration of NHC with dash lines representing the 5-95% CI. Blue dots with error bars represent observed plasma concentrations of NHC. Concentration-time profiles after administration of molnupiravir at 50 mg (a), 100 mg (b), 200 mg (c), 300 mg (d), 400 mg (e), 600 mg (f) and 800 mg (g) are shown.

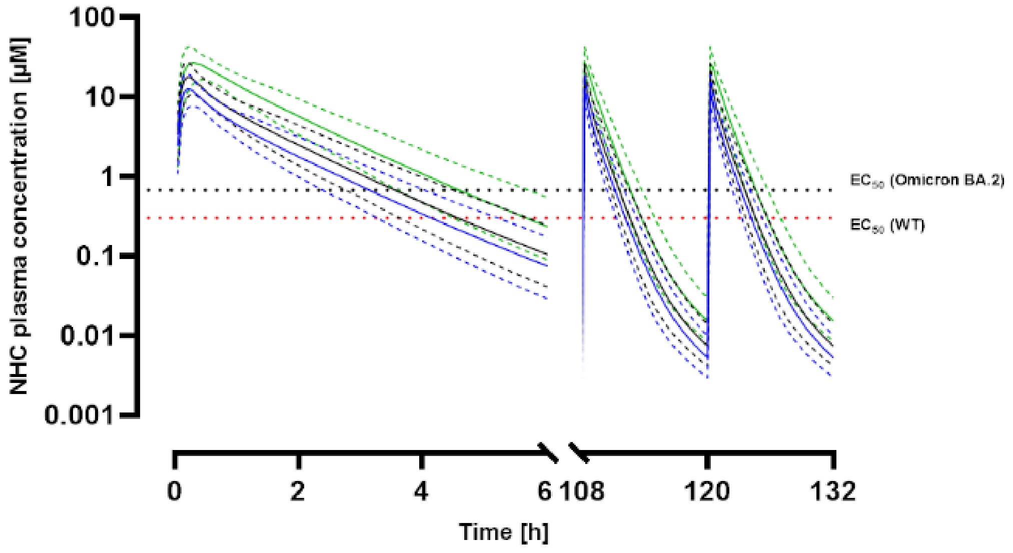

Figure S3. NHC plasma concentration-time profile for neonates at different doses

NHC plasma concentration-time profiles for 10 mg/kg BID (blue), 14 mg/kg BID (black), 28 mg/kg BID (green) doses in neonates. The continuous lines represent the predicted mean plasma concentrations, whereas the lower and upper dashed line depict 95% confidence interval. The EC_{50} for the omicron (black) variant and the wildtype (red) are shown as dashed lines.

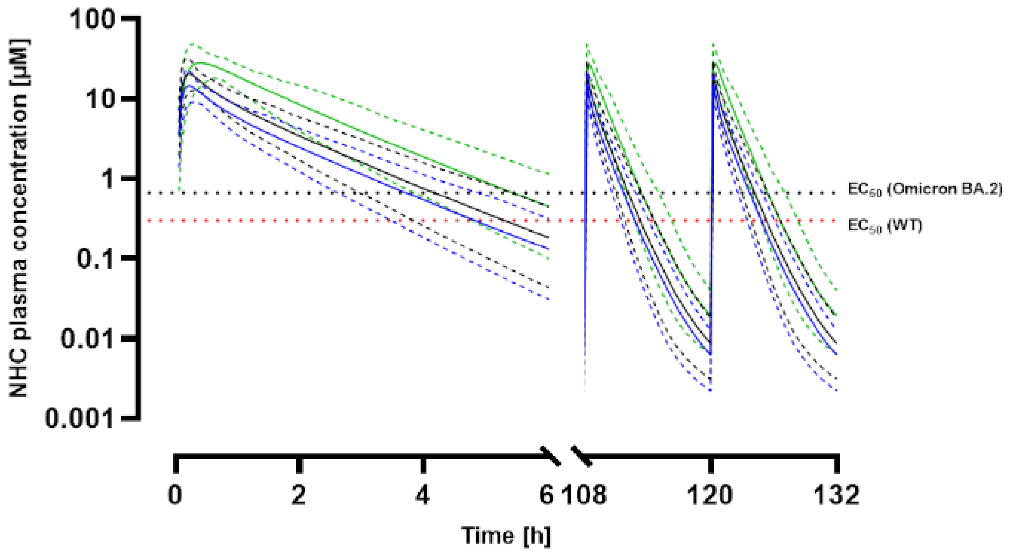

Figure S4. NHC plasma concentration-time profile for infants at different doses

NHC plasma concentration-time profiles for 10 mg/kg BID (blue), 14 mg/kg BID (black), 28 mg/kg BID (green) doses in infants. The continuous lines represent the predicted mean plasma concentrations, whereas the lower and upper dashed line depict 95% confidence interval. The EC_{50} for the omicron (black) variant and the wildtype (red) are shown as dashed lines.

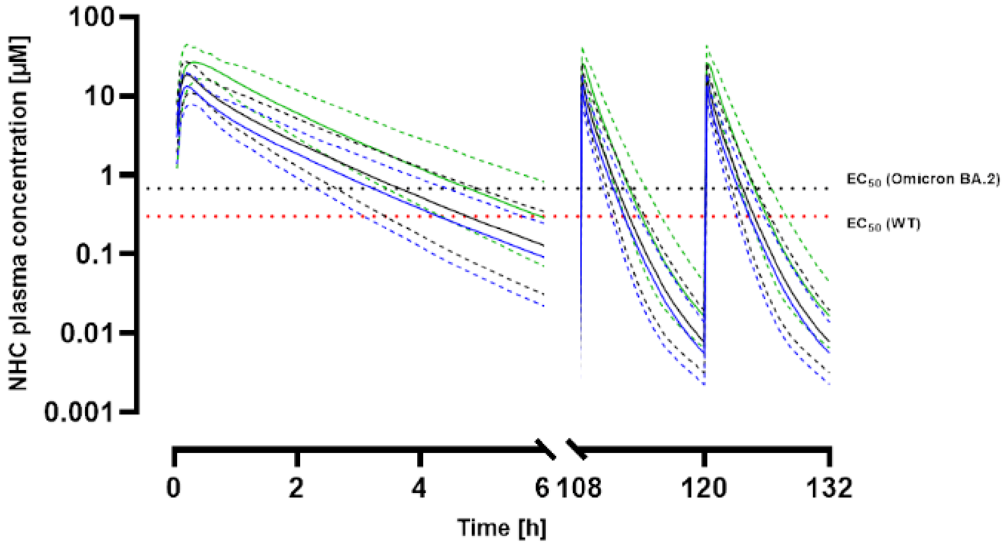

Figure S5. NHC plasma concentration-time profile at different doses for children in early childhood

NHC plasma concentration-time profiles for 10 mg/kg BID (blue), 14 mg/kg BID (black), 28 mg/kg BID (green) doses in children in early childhood. The continuous lines represent the predicted mean plasma concentrations, whereas the lower and upper dashed line depict 95% confidence interval. The EC_{50} for the omicron (black) variant and the wildtype (red) are shown as dashed lines.
